# A New Strategy to Fabricate Nanoporous Gold and Its Application in Photodetector

**DOI:** 10.3390/nano12091580

**Published:** 2022-05-06

**Authors:** Shunlin Yu, Chuan Liu, Songjia Han

**Affiliations:** 1 State Key Laboratory of Optoelectronic Materials and Technologies and Guangdong Province Key Laboratory of Display Material and Technology, School of Electronics and Information Technology, Sun Yat-Sen University, Guangzhou 510275, China; yushlin3@mail2.sysu.edu.cn (S.Y.); liuchuan5@mail.sysu.edu.cn (C.L.); 2 College of Electronic Engineering, South China Agricultural University, Guangzhou 510642, China

**Keywords:** nanoporous gold, photodetector, solution-processed technique

## Abstract

Nanoporous gold (NPG) plays an important role in high-performance electronic devices, including sensors, electrocatalysis, and energy storage systems. However, the traditional fabricating methods of NPG, dealloying technique or electrochemical reduction technique, usually require complex experimental procedures and sophisticated equipment. In this work, we reported a unique and simple method to prepare the NPG through a low-temperature solution process. More importantly, the structure of the NPG-based electrode can be further controlled by using the post-treatment process, such as thermal treatment and plasma treatment. Additionally, we also demonstrate the application of the resulting NPG electrodes in flexible photodetectors, which performs a higher sensitivity than common planar photodetectors. We believe that our work opens a possibility for the nanoporous metal in future electronics that is flexible, large scale, with facile fabrication, and low cost.

## 1. Introduction

Because of the structure of open surfaces, nanoporous (NP) material possesses a much larger surface area than bulk materials. As the ligaments of the NP materials are nanoscale, the NP materials also exhibit quantum size effect, low relative density, high energy absorption efficiency, high permeability, and quantum tunnel effect [1,2,3,4]. For instance, Cao et al. found that the nanoporous indium phosphide (InP) exhibited a strong photoluminescence peak because of the quantum size effect of the nanoporous InP structure [5]. Schrier et al. found that, at room temperature, the atoms were capable of transmitting through the barriers of nanoporous graphene due to the quantum tunneling effect [6]. Taking advantage of those promising physical and chemical properties, NP materials have been extensively explored for advanced electronic devices, such as sensors, cells, catalysis, transistors, and others [7,8,9,10]. In particular, among various NP materials (e.g., NP graphene, NP silica, and NP carbon), nanoporous gold (NPG) possesses unique structural advantages, including great biocompatibility, high corrosion resistance, abundant atomic steps, high conductivity, and surface plasmon, and it has attracted more and more attention [11,12,13,14,15]. For example, the abundant atomic steps on the surface of Au ligament not only expose more catalytically active sites but also optimize the adsorption of reaction intermediates, leading to favorable reaction pathways, which allow direct functionalization for customized surface chemistry [16].

The dealloying technique is one of the most common methods to prepare NPG [17,18]. First, a layer of alloy film, which consists of Au and other reactive metals (e.g., aluminum and silver), is deposited using electrochemical, thermal evaporation, or magnetron sputtering. Then, through selectively etching away the reactive metals from the alloy, nobler gold is left to form a continuous nanoporous framework. Recently, Xiao and coauthors reported a facile dealloying technique to construct the NPG structure. Using thiol compound-mediated chemical dealloying, the feature size of the NPG was decreased to 4 nm [19]. Electrochemical deposition is another common and effective strategy for constructing NPG [20]. Wang and coauthors reported an NP metal using modified electrochemical deposition [21]. Taking advantage of molecular self-assembly/electro-deposition, the NPG was decorated by platinum at submonolayer precision. However, these reported methods are usually proceeded by the use of complicated equipment. In particular, when scaling up the traditional process to a large area, significantly quantities of the reaction solution is required, and great quantities of poisonous waste liquid are produced, which is expensive, wasteful, and harmful to the environment.

Considered an important tool to perceive and obtain various information from light, photodetectors have greatly promoted the development of society and provided convenience for human beings. In particular, the UV photodetectors with excellent anti-interference ability, simple structure, and easy miniaturization and integration have attracted great research interest in military detection, life sciences, astronautics, etc. Indeed, in recent years, various nanomaterial-based photodetectors have been proposed as these materials can effectively improve the performance of photodetectors. For instance, Qin et al. developed a MoS_2_-CuInS_2_-AuNPs hybrid photodetector, in which the AuNPs worked as a light sensitizer to enhance the photodetector’s responsibility because of its strong plasmonic resonance [22]. Khan et al. reported ReS_2_ 2D-FETs in high mobility, in which the Co nanoparticles were deposited to generate n-doping so as to increase photocurrent [23]. In this article, we developed a ZnONWs photodetector that used AuNPs as the patterned electrode of the device, while the presented photodetectors based on the literature 2D material used nanoparticles as the additive of active layers to improve some of the photodetector’s particular performance.

Herein, we proposed a facile and environment-friendly method for preparing NPG, which was constructed with gold nanoparticles (AuNPs) solution. Additionally, through a bottom-up and additive patterning technique, a flexible and highly sensitive photodetector based on patterned NPG was achieved. The advantages of the designed photodetector are as follows: (1) the porous structure of the NPG electrode developed the efficiency of light utilization; (2) the surface plasmon resonance of nanoscale ligaments of NPG can increase the sensitivity of the sensors; (3) through low-temperature solution-phase printing techniques, the NPG can be deposited on the various plastic substrates. In summary, we provide a facile, solution-processed method to build a patterned gold film with a nanoporous structure. We believe that our work offers an effective and promising strategy for NPG in the application of future functional electronics.

## 2. Experimental Section

Materials: Gold(III) chloride trihydrate (HAuCl_4_ ∗ 3H_2_O_,_ 99.9%) and Sodium citrate (HOC(COONa)(CH_2_COONa)_2_ ∗ 2H_2_O, 99%) were purchased from Sigma-Aldrich (Taufkirchen, Germany). Zinc oxide nanowires (ZnONWs, D: 50–120 nm, L: 5–50 μm) were purchased from XFNANO Materials Tech Co., Ltd. (Nanjing, China). Perfluoro (1-butenyl vinyl ether) polymer (Cytop, CLT-809M) and solvent (CT-SOLV180) were obtained from AGC Chemicals Europe (Lancashire, UK). 

Synthesis of AuNPs: The AuNPs were synthesized by Turkevich–Ferns method [24]. First, 0.08 g HAuCl_4_ was dissolved in 400 mL deionized (DI) water and stirred at 900 rpm for 5 min to obtain a light-yellow solution. After heating the solution to 100 °C, 40 mL sodium citrate solution (20 mM) was added. The solution gradually turned from light yellow to colorless, blue-black, and purplish-red. Then, we heated the purplish-red solution for 30 min to make sure the reaction was complete. To increase the concentration of AuNPs solution, the product was centrifugated (8000 rpm) for 45 min. After removing the supernatant, the black liquids stuck to the bottom of containers were collected, which were the obtained AuNPs solution with a concentration of 25 mM. The solvent of the AuNPs solution was water. 

Preparation of patterned NPG electrode: First, a glass substrate (2 × 2 cm^2^) was ultrasonically cleaned with acetone, ethanol, and DI water for 20 min, respectively. After drying the substrate with nitrogen, Cytop solution (V_Cytop_:V_sol_ = 1:10) was spin-coated at 3000 rpm for 30 s. Then, the sample was heated at 100 °C for 10 min to remove the solvent, and a layer of hydrophobic film was formed on the substrate. Subsequently, the substrate was covered with a shadow mask and treated by a plasma cleaner at 60 W for 90 s. To obtain a nanoporous electrode, the resulting AuNP solution was deposited on the substrate by spin coating (500 rpm, 30 s). Then, the sample was heated at 100 °C for 10 min to remove the solvent. Finally, we obtained an AuNP electrode with a channel length of 500 μm and a width of 1000 μm. Figure S1a shows the schematic diagram of the formation process of the AuNP electrode, and Figure S1b shows the SEM images of the AuNP electrode before post-treatment.

Preparation of flexible photodetector: First, the AuNP electrode was deposited on a flexible substrate, Polyethylene naphthalate two formic acid glycol ester (PEN), using the above method. Secondly, 3 mg ZnONWs were dispersed in 10 mL of ethanol and water mixed solution (V_ethanol_:V_water_ = 2:1) and ultrasonically dispersed for 10 min. A milky white ZnONWs dispersion was obtained. Then, the ZnONWs dispersion (0.5 μL) was dropped on the channel of NP electrodes and heated at 100 °C for 10 min. Finally, two silver wires were attached to both ends of the electrode with conductive silver paste for photoelectrical measurement. 

Characterization of the device: The microstructure of the NPG electrode was characterized using field emission scanning electron microscopy (SEM, Carl Zeiss (Jena, Germany), SUPRA 6) and optical microscopy (Carl Zeiss, Axio CSM 700). The distribution of AuNP size was analyzed via high-resolution transmission electron microscopy (TEM, FEI titan3 G2 60-300 (Hillsboro, OR, USA)). A digital multimeter (Kethley (Beaverton, OR, USA), 2400) was used to measure the current change of the photodetector. A laser diode with a wavelength of 380 nm was used as the light source. The absorbance of the sample was analyzed by an ultraviolet spectrophotometer with a wavelength from 300 nm to 800 nm (Thermo (Waltham, MA, USA), Evolution 201). The contact angle was tested using a contact angle measuring instrument (DataPhysics, OCA 15ec, San Jose, CA, USA). X-ray diffraction pattern (XRD) was characterized using an X-ray diffractometer (Empyrean) (PANalytical, Almelo, The Netherlands).

## 3. Results and Discussion

**Figure 1:**
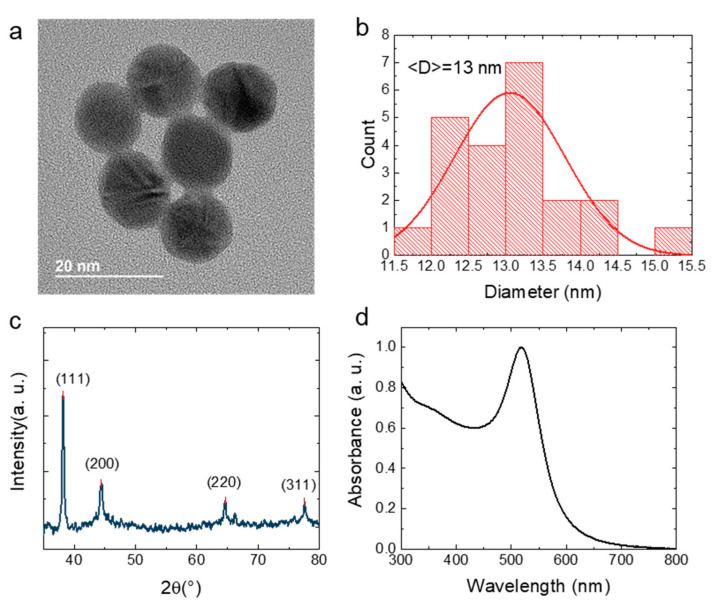
(**a**) TEM image of the AuNPs. (**b**) Size distribution of the AuNPs. (**c**) XRD spectrum of the AuNPs. (**d**) Ultraviolet–visible (UV-Vis) absorption spectrum of the AuNPs solution.

The shapes of the AuNPs were characterized by TEM. As shown in Figure 1a,b, the synthesized AuNPs possess a smooth spherical structure and have an average diameter of 13 nm. Figure 1c shows the XRD spectrum of the AuNPs. Four obvious characteristic peaks were observed at 2θ = 38.1, 44.2, 64.6, and 77.5°, which correspond to the (111), (200), (220), and (311) planes, respectively, in the fcc gold lattice [25]. Because of the localized surface plasmon resonance of the metal nanoparticles, the gold nanoparticles usually exhibit obvious extinction peaks in the UV–Vis range. Additionally, the optical performances of the gold nanomaterials would be affected by their shape and size [26,27]. According to the research of Chen and his coauthors, the sphere-shaped Au nanoparticles displayed only one surface plasmon peak. Conversely, two major surface plasmon peaks would be found in the absorption spectra of Au nanorods [28]. Figure 1d depicts the absorption spectrum of the AuNPs solution; only one surface plasmon wavelength appears at 518 nm. Thus, we suggest that the shape of the AuNPs is nanosphere.

**Figure 2:**
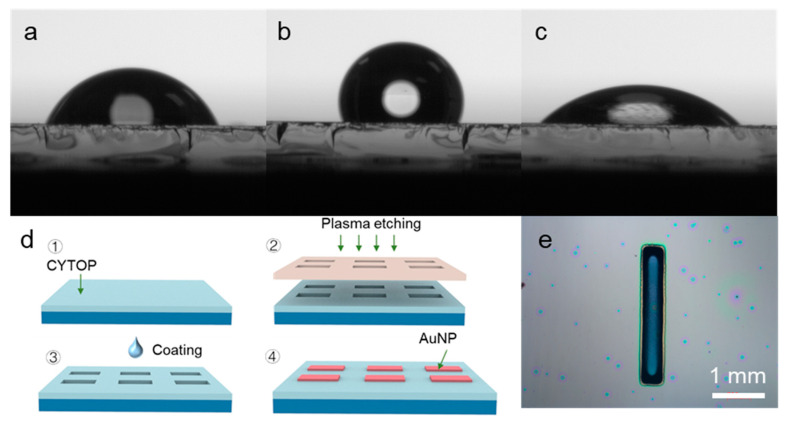
Contact angle images of the glass substrate at the different stations: (**a**) untreated, (**b**) coating Cytop, (**c**) after plasma treatment. (**d**) Schematic illustration of the fabrication of the patterned NPG electrodes. (**e**) Optical microscope image of the patterned NPG electrode.

Figure 2a–c show the contact angles of the glass substrate. Without any treatment, the contact angle of the bare glass was 66.8°. When the glass substrate was deposited with a layer of Cytop film, its contact angle was increased to 108.5°. The surface of the glass was changed from hydrophilic to hydrophobic, on which the aqueous solution was difficult to spread. Then, removing the Cytop film by plasma etching, the contact angle of the substrate decreased to 43.5° because a large number of active groups were grafted on the surface of the glass substrate. Figure 2d shows the detailed fabrication process for patterning NPG electrodes. Because of the change in surface hydrophilicity of the substrate, when spin-coating the AuNPs solution, the aqueous solution was only wetted in the hydrophilic region and was dewatered in the hydrophobic region. Thus, a specific patterned NPG electrode was formed, as shown in Figure 2e [29]. 

**Figure 3:**
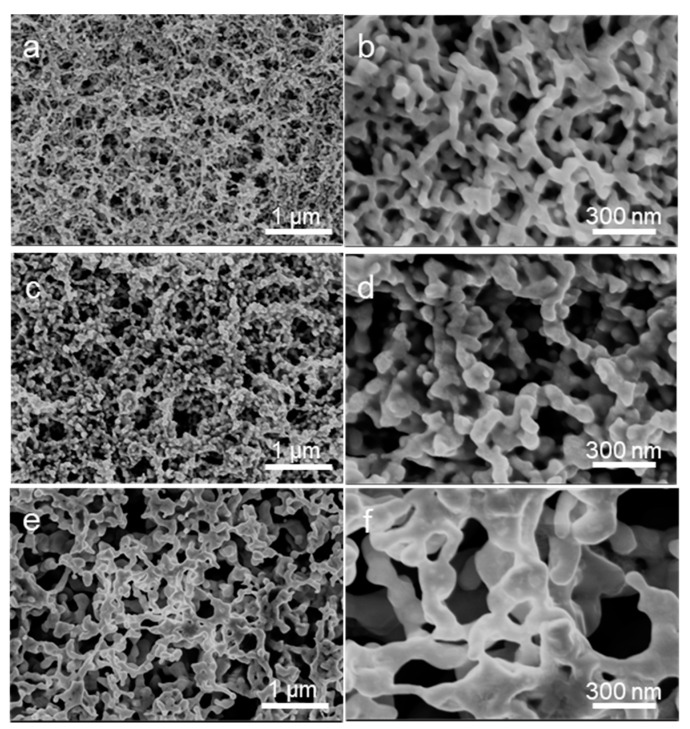
SEM images of the NPG electrode annealed at different temperatures: (**a**,**b**) 200 °C, (**c**,**d**) 300 °C, (**e**,**f**) 400 °C.

The microstructure of the NPG electrode was characterized by SEM, as shown in Figure 3. Since the melting point of AuNPs is much lower than that of bulk gold, when the NPG electrode was annealed at 200 °C, electron exchange occurred in the interface of two adjacent AuNPs; the adjacent AuNPs presented elastic deformation and formed a sintering neck [30]. As a result, the average ligament thickness of the porous electrode is about 38.1 nm. When increasing the sintering temperature to 300 °C, a lot of gold atoms on the surface of AuNPs were able to diffuse towards the sintering neck, the sintering neck between the adjacent AuNPs grew, the skeleton of the porous electrode increased gradually, and the average ligament thickness of porous electrode increased to 75.8 nm. When we annealed the sample at a higher temperature of 400 °C, the AuNPs began to melt, and the sintering neck increased quickly. Finally, the skeleton of the NPG electrode increased to 98.2 nm. 

**Figure 4:**
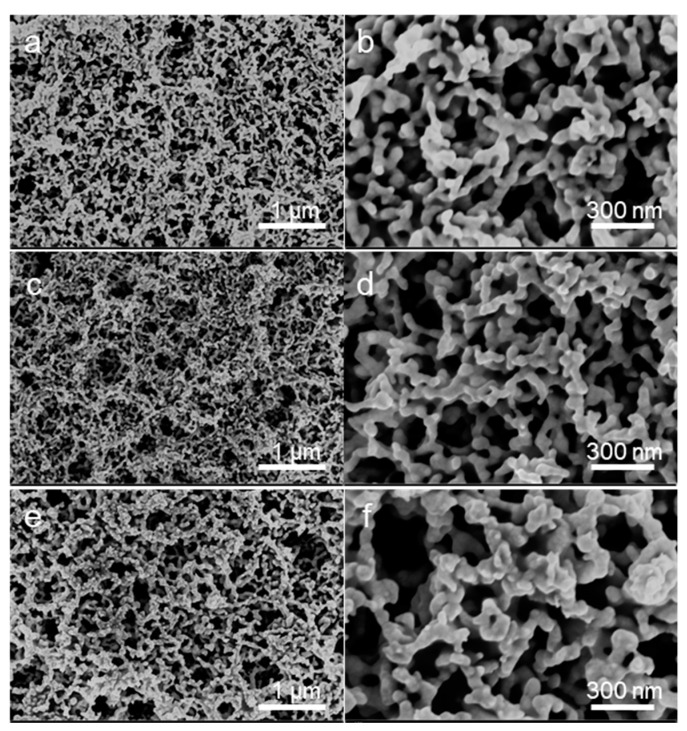
SEM images of the NPG electrode treated with plasma with different powers: (**a**,**b**) 60 W, (**c**,**d**) 70 W, (**e**,**f**) 80 W. Dry air was supplied during the plasma treatment.

Furthermore, because the plastic substrates easily suffer from deformation when annealed at high temperatures, we also studied the effect of nonthermal treatment on the structure of the NPG electrodes. Figure 4 displays the SEM images of the NPG electrodes treated by plasma with different power. It can be observed that when the samples were treated with plasma with 60% and 70% output power, the obtained porous structures were even and regular and possessed average widths of the ligaments of 39.8 nm and 38.5 nm, respectively. When increasing the outpower to 80%, more Joule heat was generated and led to the AuNPs melt agglomeration. As shown in Figure 4e,f, the ligament width of the porous electrodes was increased to 75.5 nm. Hence, the structure of the NPG electrode can be further controlled by plasma or thermal treatment. 

**Figure 5:**
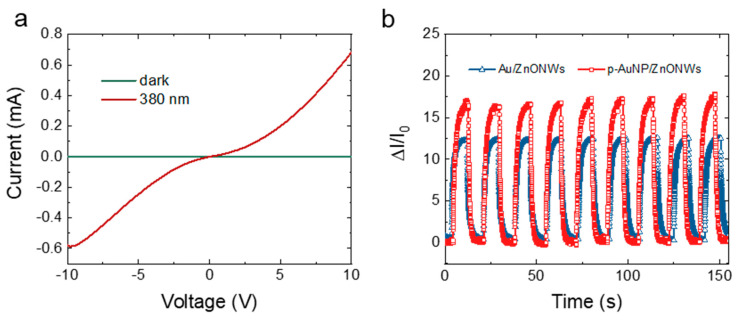
(**a**) I−V curves of the ZnONWs-based photodetector under dark and light, the applied bias voltage was 5 V. (**b**) Relative change in the current curves of the photodetectors.

Figure 5a displays the I–V curves of the ZnONWs-based photodetector under dark and light illumination conditions, respectively. The optical picture of the photodetector based on the NPG electrode is shown in Figure S2. Without illumination, the surface of ZnONWs exhibited a number of defects; the electrons in the ZnONWs were captured by the oxygen adsorbed on the ZnONWs surface [31,32]. Therefore, the ZnONWs possessed high resistance, and the dark current of the device was very small. When applying a bias voltage of 5 V, the dark current of the device was 1 μA. Once the ultraviolet (UV) illumination was applied, the electrons located in the valence band of ZnONWs could absorb the energy of photons and transit from the valence band to the conduction band [33]. With the concentration of electron carriers increasing, the conductivity of the photodetector was enhanced. Figure 5b shows the relative change in the current (ΔI/I_0_) of the photodetectors. Compared with the device based on a normal planar Au electrode, it is clear that the ΔI/I_0_ of the NPG-based photodetector raises more sharply, indicating a higher sensitivity. Since response time is an important property of the photodetector, the rising and recovery process of the device were plotted in Figure S3a,b. Our photodetector showed a rise time of 3.5 s, which was the photocurrent increased from 10% to 90% of the maximum. Most notably, the recovery time of the photodetector (photocurrent dropped from 90% to 10% of the maximum) based on the NPG electrode decreased to 1.47 s, while the response time and recovery time of the normal Au/ZnONWs device was 3.43 s and 3.23 s, respectively (in Figure S4). We speculate that, compared with the smooth surface of normal gold films, the NPG electrode was constructed with nanoparticles, in which the presence of nanoparticles would destroy the ordered state of the electrode surface. The emergence of energy traps can promote the recombination of electrons and holes [33]. We suggested that our NPG electrode can improve the sensitivity of the photodetector because of the following: (1) Owing to the surface plasmonic effects of the AuNPs, which are the ligaments of the NPG electrode, a strong electric field is generated near the surface of NPG electrode when the ultraviolet light was absorbed by ZnONWs. The generated electric field near the PNG electrode can attract the photogenerated electrons in ZnONWs. As a result, the photogenerated electrons will quickly separate from holes and transfer to the NPG electrode. Thus, the transit time of electrons from ZnONWs to the NPG electrode is reduced. The photo gain in the photodetector can be calculated by the following equation [34,35,36]:

(1) $$G=\frac{\tau_{n}\left(\mu_{n}+\mu_{p}\right)E}{L}=\frac{\tau_{n}}{\tau_{t}}\left(1+\frac{\mu_{p}}{\mu_{n}}\right)$$

where $\tau_{t}$ is the transit time for minority electrons to transport between the contact electrodes of the device, $\tau_{n}$ is the carrier lifetime, $\mu_{n}$ and $\mu_{p}$ are electron and hole mobility, respectively [36]. (2) According to Marcus’ theory, nano-sized metal particles can be used as antennas to absorb and concentrate the light in plasmon resonance. Then, the absorbed light energy can be converted into hot carriers in semiconductor materials through a coherent process, which means that the hot electrons generated in metal are transmitted to the interface and to the semiconductor layer through the energy barrier. Therefore, under light illumination, the electrons in the NPG electrode are affected by photon energy and rise from below the Fermi level to form hot electrons. Then, some of these generated hot electrons will transfer to the metal/semiconductor interface. When the kinetic energy of these hot electrons reaching the interface exceeds the potential barrier, they can transit through the Schottky barrier to the conduction band of the ZnONWs. Therefore, we suggest that the electromagnetic field induced by the local surface plasmon resonance (LSPR) of the AuNPs, increases the number of hot electrons at the interface, which can increase the photocurrent of the device [34,37,38,39].

**Figure 6:**
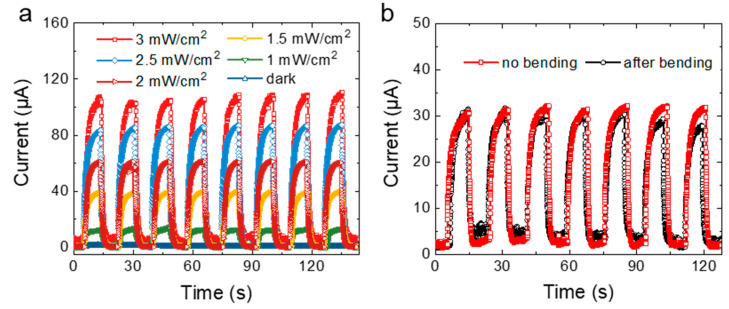
(**a**) Photocurrent of the NPG/ZnONWs-based photodetector under different incident light intensity, (**b**) photocurrent of the NPG/ZnONWs-based photodetector before and after 1000-times bending.

We also studied the dynamic response of the NPG-based photodetector to UV light with different intensities (Figure 6a). The applied bias voltage was 5 V. Without illumination, because of the oxygen adsorption and energy band bending of the ZnONWs, the ZnONWs film performed a high-resistance state, and the dark current of the device was very small. When applying UV light illumination with 1 mW/cm^2^, the photocurrent increased to 12 μA. Then, the intensity of light illumination was increased to 1.5 mW/cm^2^, 2 mW/cm^2^, 2.5 mW/cm^2^, and 3 mW/cm^2^, respectively; as expected, the photocurrent of the device increased to 39.83 μA, 61.64 μA, 86.39 μA, and 107.37 μA as well. The sensitivity of the photodetector can be calculated by the equation:

(2) $$s\left(\%\right)=\frac{I_{light}-I_{dark}}{I_{light}}\cdot 100$$

where $I_{light}$ and $I_{dark}$ are the currents under UV light and dark [40,41]. When increasing the light illumination to 3 mW/cm^2^, the sensitivity of the photodetector was 7 × 10^3^% under UV light. Additionally, the photodetector based on the NPG electrode also performed good reproducibility and repeatability during the light on-off cycling. Then, to demonstrate the flexibility of the device, the sample was fixed on a homemade motorized moving stage and subjected to bending 1000 times. The bending radius of the photodetector was 3 mm, as shown in Figure S5. Then, we collected the photocurrents of the devices before and after the bending test. As shown in Figure 6b, two overlapping curves demonstrate that the device has a great flexibility.

## 4. Conclusions

In summary, we have successfully prepared the NPG electrode based on gold nanoparticles. Compared with traditional dealloying and electrochemical deposition techniques, our method is simple, without toxic byproducts, and environmentally friendly. The morphology of the NPG electrode can be controlled by a simple post-treatment. Additionally, because of the low-temperature solution routine, the photodetector was fabricated on a flexible plastic substrate. Taking advantage of the nanostructure of the NPG electrode, which possesses surface plasmonic effects and local surface plasmon resonance, the photodetector based on the NPG electrode displayed higher sensitivity than the traditional planar electrode. After 1000-times bending tests, the device still worked well and performed with excellent flexibility. Besides the photodetector, we suggest that the NPG electrode also can be utilized in other sensors, such as strain sensors, temperature sensors, and chemical sensors, which open an avenue for the fabrication of future high-performance, low-cost, and flexible sensors.

## Data Availability

The data presented in this study are available in this article.

## Supplementary Materials

The following supporting information can be downloaded at: https://www.mdpi.com/article/10.3390/nano12091580/s1. Figure S1: (a) The schematic diagram of the formation process of the AuNP electrode. (b) SEM images of the AuNP electrode before posttreatment; Figure S2: Optical picture of the photodetector based on NPG electrode; Figure S3: The corresponding I-t curves of the photodetector based on NPG electrode. (a) The enlarged rise process of the current response under UV illumination, (b) The enlarged recovery process of the current response under UV illumination; Figure S4: The corresponding I-t curves of the Au/ZnONWs photodetector. (a) The enlarged rise process of the current response under UV illumination, (b) The enlarged recovery process of the current response under UV illumination; Figure S5: The schematic diagram of the bending test.
