# Supplementary material for: A New Strategy to Fabricate Nanoporous Gold and Its Application in Photodetector
Source: Nanomaterials (Basel). 2022 May 6;12(9):1580. doi: 10.3390/nano12091580 (PMC9102659; doi:10.3390/nano12091580)
Supplement: Supplementary file 1 [file nanomaterials-12-01580-s001.zip › nanomaterials-1661708-supplementary.pdf]

Supporting Information

A New Strategy to Fabricate Nanoporous Gold and Its Application in Photodetector

Shunlin Yu [1], Chuan Liu [1] and Songjia Han [2,*]

[1] State Key Laboratory of Optoelectronic Materials and Technologies and Guangdong Province Key Laboratory of Display Material and Technology, School of Electronics and Information Technology, Sun Yat-Sen University, Guangzhou 510275, China; yushlin3@mail2.sysu.edu.cn (S.Y.); liuchuan5@mail.sysu.edu.cn (C.L.)

[2] College of Electronic Engineering, South China Agricultural University, Guangzhou 510642; China.

* Correspondence: hansongjia@scau.edu.cn

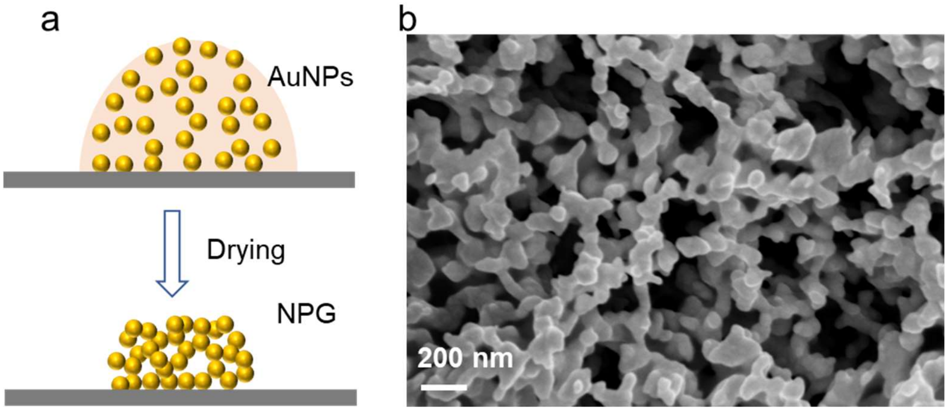

Figure S1. (a) The schematic diagram of the formation process of the AuNP electrode. (b) SEM images of the AuNP electrode before posttreatment.

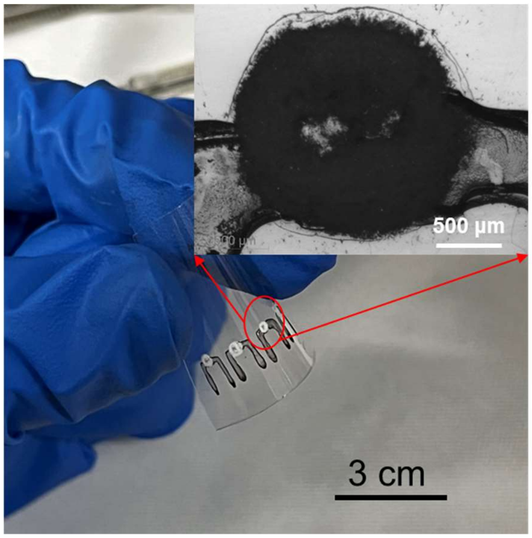

Figure S2. Optical picture of the photodetector based on NPG electrode.

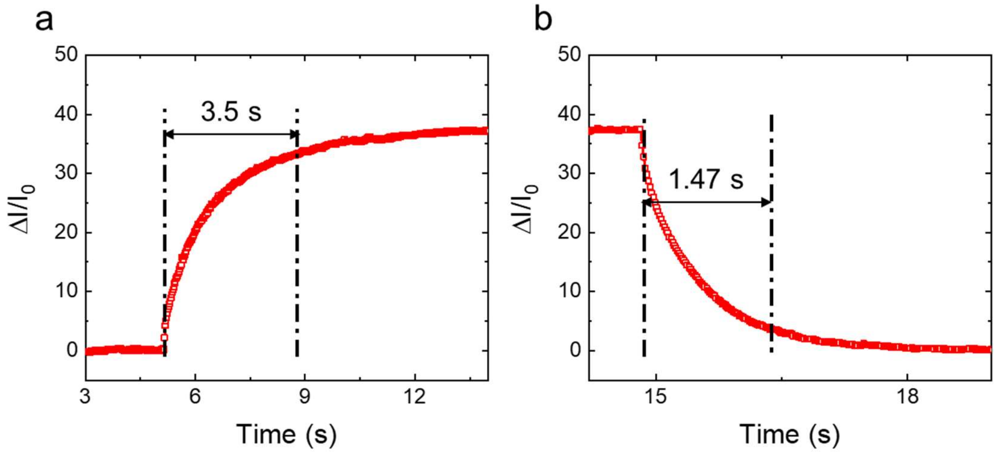

Figure S3. The corresponding I-t curves of the photodetector based on NPG electrode. (a) The enlarged rise process of the current response under UV illumination, (b) The enlarged recovery process of the current response under UV illumination.

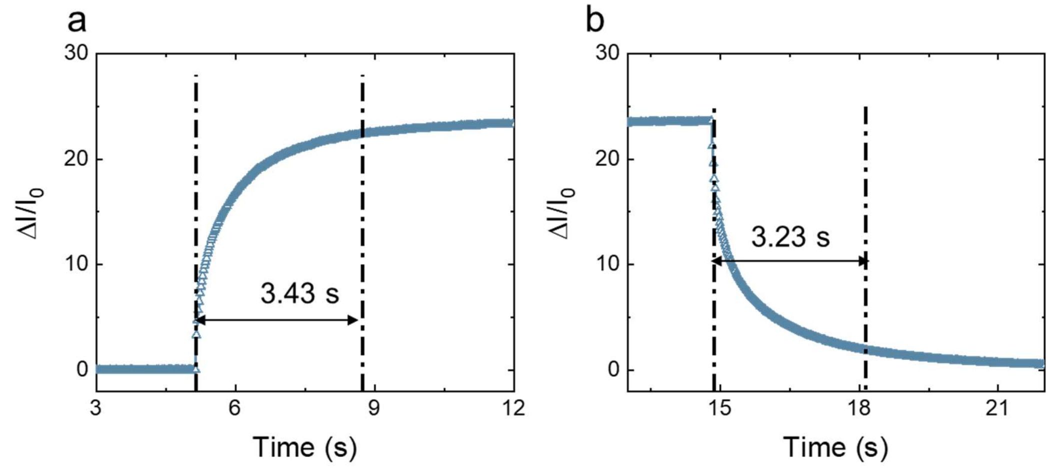

Figure S4. The corresponding I-t curves of the Au/ZnONWs photodetector. (a) The enlarged rise process of the current response under UV illumination, (b) The enlarged recovery process of the current response under UV illumination.

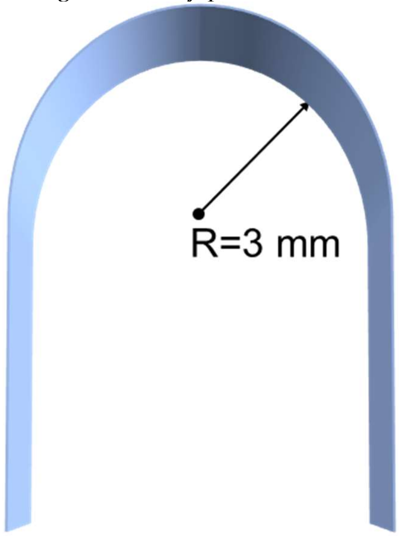

Figure S5. The schematic diagram of the bending test.
